# Haemophagocytic lymphohistiocytosis during pregnancy: a case presentation and literature review

**DOI:** 10.1515/crpm-2021-0004

**Published:** 2022-03-04

**Authors:** Larissa Fávero Vanraes, Veerle Beckers, Kim Van Berkel, Leonardo Gucciardo, Gilles Faron

**Affiliations:** Department of Gynaecology and Obstetrics, Vrije Universiteit Brussel, Brussels Health Campus, Brussels, Belgium; Division of Haematology, Department of Internal Medicine, Vrije Universiteit Brussel, Brussels Health Campus, Brussels, Belgium; Department of Medical Genetics, Vrije Universiteit Brussel, Brussels Health Campus, Brussels, Belgium; Division of Prenatal Medicine and Obstetrics, Department of Gynaecology and Obstetrics, Vrije Universiteit Brussel, Brussels Health Campus, Brussels, Belgium

**Keywords:** anhydramnios, haemophagocytic lymphohistiocytosis, pregnancy

## Abstract

**Objectives:**

Haemophagocytic lymphohistiocytosis (HLH) is a potentially fatal disorder of the immune system that typically occurs in the paediatric population. Diagnosing this rare disease in the adult population is challenging, particularly during pregnancy.

**Case presentation:**

We present a case of a gravid patient developing HLH at week 13 of gestation undergoing a medical termination of pregnancy at 27 weeks due to anhydramnios and associated stopped foetal growth.

**Conclusions:**

Disease triggers could vary from a simple viral infection to the pregnancy as such causing the disorder. Treatment should benefit the mother and limit the foetal harm.

## Introduction

Haemophagocytic lymphohistiocytosis (HLH) is a disorder of the immune system in which hyperactivation of macrophages and T-lymphocytes leads to uncontrolled cytokine storm, causing the hyperinflammatory state characteristic of this syndrome [1]. This over proliferation and infiltration of histiocytes manifests as acute illness with prolonged fever, cytopenia and hepatosplenomegaly [2]. Other associated findings are hypertriglyceridemia, coagulopathy with hypofibrinogenemia, haemophagocytosis and elevated ferritin [3]. The mortality rate ranges from 26.5% to 74.8% according to studies and etiologies [4].

HLH can be genetically determined, commonly referred to as Primary HLH or Familial Haemophagocytic Lymphohistiocytosis (FHL). The secondary form is mainly caused by environmental factors such as infections, oncologic or autoimmune diseases [1]. To distinguish these two different conditions can be difficult, since infections are usually the trigger of the primary form. FHL is inherited in an autosomal recessive (AR) way. Classical presentation occurs after contact with a trigger such as a viral infection and can lead to the patient death by uncontrolled inflammation, multiple organ failure or secondary severe infection if the disease is left untreated [5]. These primary HLH are generally diagnosed in very young patients below one year of age [6]. Late onset is rare but has been described [7]. The diagnosis of FHL relies on molecular genetic testing [1, 2]. In that case, five disease subtypes have been described: FHL1, FHL2, FHL3, FHL4 and FHL5. In which pathogenic variants of four genes have been identified to be causative: *PRF1 (FHL2), UNC13D (FHL3), STX11(FHL4) and STXBP2 (FHL5)* [2]

The diagnosis of HLH can be challenging. There have been only few cases of HLH reported during pregnancy. Considering the potential serious effects to the mother and foetus, it is very important to avoid delay in recognizing this condition, because early treatments may clearly improve outcomes. We present a case of HLH that provides clinicians insight into the outcome and pathophysiology of this rare disease.

## Case presentation

A 26-year-old woman, gravida 1 para 0, 13 week pregnant at that time and no significant comorbidities, presented with fever, general unwellness, dry cough and muscle aches. Paracetamol and empiric antibiotherapy with amoxicillin had already been started by the general practitioner, but due to persistent pyrexia the patient was referred to the emergency room.

At the time of admission, her vital signs were as follows: body temperature of 38.5 °C, blood pressure 127/70 mmHg, heart rate of 113 beats per min (BPM) and oxygen saturation of 97% while breathing ambient air. Physical examination showed abdominal bloating, diffuse abdominal pain mostly in the epigastric and suprapubic regions and no significant peripheral adenopathy. Laboratory tests indicated striking liver function disturbance (aspartate-aminotransferase (ASAT) 120 U/L, alanine-aminotransferase (ALAT) 54 U/L, gamma-glutamyl transferase (GGT) 124 U/L and hyperbilirubinemia of 1.42 mg/dL with prolonged activated partial-thromboplastin time (aPTT), pancytopenia (platelets of 108.000/L; white blood cell count of 3.300 with lymphocyte count of 420/mm^3^ and hemoglobin of 10.9 g/dL), mild hypertriglyceridemia (196 mg/dL), elevated levels of lactate dehydrogenase (LDH) 1736 U/L, elevated C-reactive protein (177.6 mg/dL), unmeasurable haptoglobin and elevated ferritin (2,898 mcg/L). Blood gas analysis showed respiratory alkalosis. X-ray revealed right basal pleural effusion and a possible lung infiltrate could not be excluded. Initially a diagnosis of community-acquired pneumonia (CAP) was considered. The antibiotic therapy was changed to intravenous Amoxicillin-clavulanic-acid and the patient was admitted to the department of pneumology.

Legionella urinary antigen, nasopharyngeal swab for influenza testing as well as sputum culture were negative. Serological testing for Mycoplasma infections and Human Immunodeficiency Virus (HIV) were also negative. Hepatitis serology indicated no current or recent infection with hepatitis A, B nor C. Although CMV PCR could not be performed due to local reimbursement criteria, repeated CMV serology showed no seroconversion. EBV PCR was negative. Since this event occurred 1 year before the COVID-19 pandemic, this diagnosis can be excluded.

Radiology exams such as abdominal ultrasound and magnetic resonance (MRI) were requested with the latter demonstrating hepatosplenomegaly with ascites. A prenatal ultrasound confirmed a normal evolutive pregnancy of 13 weeks. A multidisciplinary consultation meeting (haematologists, pneumologists, obstetricians, geneticians and neonatologists) discussed results. Since pancytopenia and ferritin levels were not so worrying, it was initially opted to not perform a bone marrow aspiration in order to support the diagnosis of haemophagocytic lymphohistiocytosis. Additional testing to exclude malignancy was performed such as thoracocentesis for pleural fluid analysis and protein electrophoresis. Auto-immune testing, consisting of Antinuclear antibody (ANA and Antineutrophil cytoplasmic antibody (ANCA) screening, was negative. Antibodies for antiphospholipid syndrome (APS) were also analysed since cases of HELLP syndrome combined with APS before 20 weeks gestation have been reported in the literature [8]. Four days after the patient’s admission, a bone marrow aspiration and biopsy was finally done due to progressive pancytopenia and hyperferritinemia. The myelogram revealed important haemophagocytosis confirming the diagnosis of haemophagocytic lymphohistiocytosis. A secondary HLH due to viral infection was presumed initially since leukopenia with lymphopenia was present. Treatment with solumedrol 125 mg i/v followed by 40 mg dexamethasone per os was started with favourable clinical evolution. After a 10-day stay in the hospital the patient was discharged and further follow-up via haematology and obstetrics was foreseen. Dexamethasone 40 mg was continued two weeks and was then tapered with 10 mg daily.

**Figure 1:**
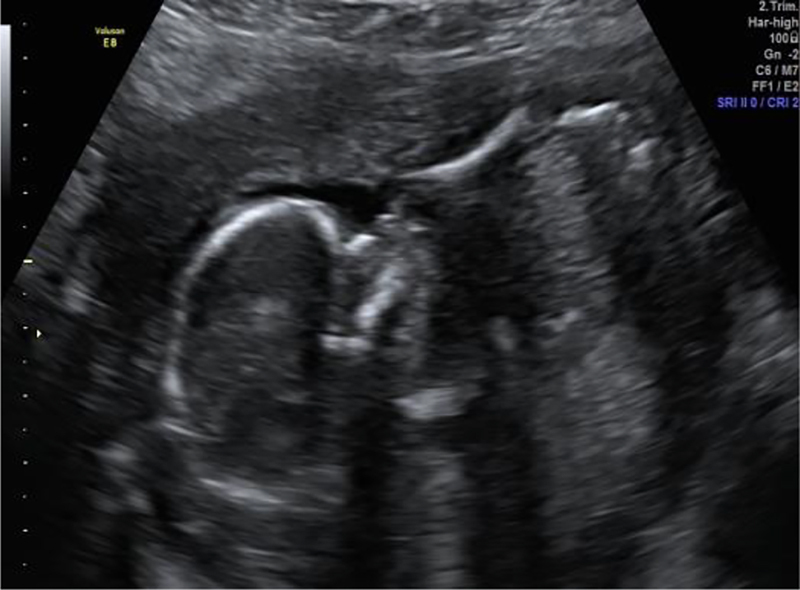
Ultrasound at 18 weeks showing anhydramnios.

**Figure 2:**
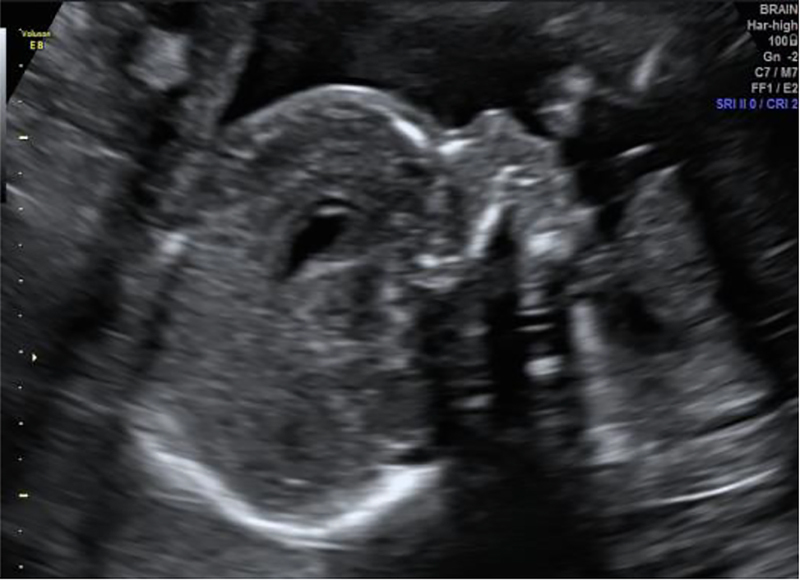
Ultrasound at 21 weeks revealing a significant improvement of amniotic fluid index (oligohydramnios).

At 18 weeks and 4 days of gestation, the patient was admitted to the Maternal Intensive Care for a short 2-day stay. The purpose of her admission was to investigate a recently developed hypertension within the scope of severe intrauterine growth restriction (IUGR). Foetal ultrasound at 18 weeks had shown anhydramnios, extreme IUGR with an estimated weight below the 1st percentile (Figure 1) and intermittent absent end-diastolic flow in the single umbilical artery doppler. Blood pressure at the time of her admission was 161/100 mmHg and no signs of pre-eclampsia were present except traces of proteinuria, diagnosed by a dipstick test. At the time she was taking 10 mg dexamethasone daily. The protein/creatinine ratio was 0.16 and laboratory routine testing showed no significant abnormalities. Because a previous foetal ultrasound at 14 weeks and 6 days was shown to be normal and maternal safety was assured, the option of therapeutic termination of pregnancy was not proposed right away. In attempting to account for the possibility of resolution of anhydramnios as steroid therapy was being tapered, a conservative policy was chosen. And indeed, the obstetric ultrasound at 20 weeks revealed a significant improvement of amniotic fluid index even if it was still described as oligohydramnios (Figure 2). However, two weeks after treatment with dexamethasone was terminated, the mother was readmitted with signs of disease activity as she reported to have had recurrent fever. Laboratory results showed elevated levels of LDH (1307 U/L), C-reactive protein (97.2 mg/L) and ferritin (1797 μg/L). A bone marrow aspiration was repeated and myelogram confirmed recurrence of haemophagocytosis. Treatment with dexamethasone 20 mg in association with ciclosporin (target 200–300 μg/L) was offered and favourable maternal response was observed. Dexamethasone was then tapered every two weeks. An adult-onset presentation of the hereditary form was to be ruled out in a next phase.

**Figure 3:**
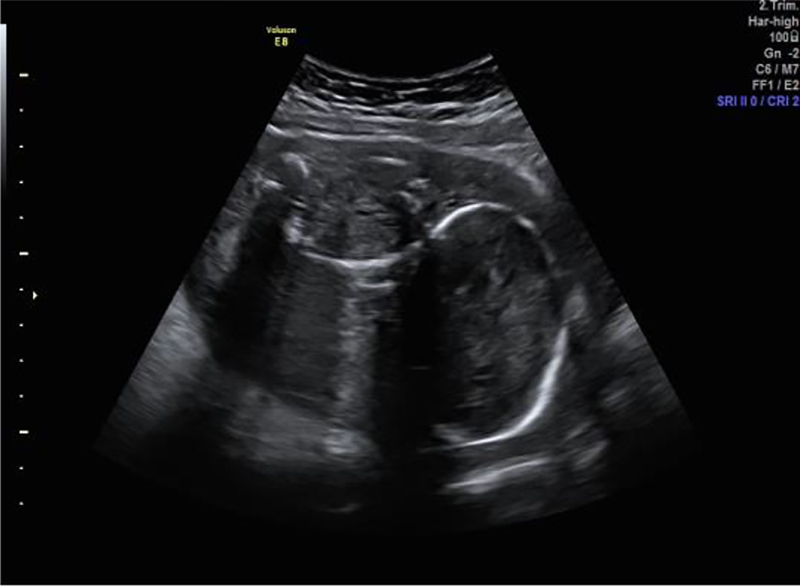
Ultrasound at 27 weeks showing a stopped foetal growth due to chronic anhydramnios.

**Figure 4:**
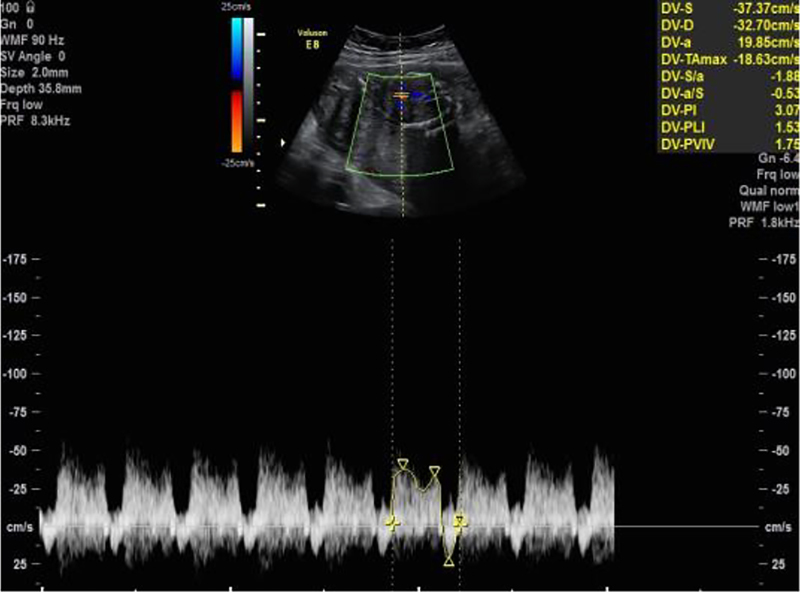
Ultrasound at 27 weeks showing a reversed a-wave in ductus venosus.

A foetal MRI of the foetus confirmed oligohydramnios and revealed foetal hepatosplenomegaly. Weekly obstetrical ultrasound controls confirmed severe oligohydramnios and extreme IUGR with a EFW of 285 g at 23 weeks and 4 days of gestation. Later, further ultrasound controls showed progressively anhydramnios and associated stopped foetal growth (EFW 272 g at 27 weeks and 1 day) with abnormal dopplers (Figures 3 and 4). As a result, due to very poor prognosis, the patient was recommended to terminate her pregnancy. She underwent medical termination of pregnancy at 27 weeks and 5 days of gestation with prompt expulsion of a nonviable female foetus with a weight of 259 g.

Anatomopathological examination supported placental insufficiency by describing thrombosis of the umbilical artery and multiple areas of placental infarction. Furthermore, foetal autopsy confirmed severe growth restriction as the measurements corresponded to a 20 weeks foetus. Surprisingly histology of foetal liver and spleen revealed signs of haemophagocytic syndrome. Such findings could possibly suggest genetic underground. The fact that both mother and foetus were affected suggests either a passage of toxicity through the placenta, with secondary inflammation activation reaction in the foetus or the possibility of FHL at the condition that the father would have to be a healthy carrier. However the probability is really weak as the incidence of FHL is 1/50,000.

The patient continued to be followed up by haematologists. Cyclosporine was tapered after 6 months of treatment and no signs of reactivation were observed after complete withdrawal. Advice was given to avoid pregnancy until one year after cyclosporine was stopped. Molecular genetic testing by conventional sequencing for presence of *STXBP2* mutation (association with Primary HLH and adult onset – autosomal recessive, homozygous or heterozygous defect) was performed in the patient. No genetical mutations could be identified in this gene.The patient had an uncomplicated pregnancy and delivered a healthy baby two years after termination of immunosuppressive treatment.

## Discussion

This presented case of HLH illustrated the difficulties in diagnosing this rare condition in pregnant woman. Although initially the patient fulfilled at least five of the eight criteria established by the Histiocyte Society for the diagnosis of HLH [3] – 1-prolonged fever >7 days; 2-splenomegaly; 3-hypertriglyceridemia; 4-haemophagocytosis and 5-hyperferritinemia – the diagnosis of pneumonia with a possible underlying pathology compromising the immune system seemed to be a more reasonable diagnosis at first, given the rarity of cases of HLH. Thrombopenia, despite its presence, was not severe enough to fulfil the HLH criteria as the platelets count was above 100.000/L and the patient was initially only slightly anaemic. Using the H-score, a first validated score devoted to the diagnosis of secondary HLH and thus more suitable to the adult population [9], a score of 248 was shown which gives a 99.32% probability of having HLH.

Immunophenotyping (blood test) confirmed lymphopenia with low T- and NK cell count and normal NK-cell activity with high plasma concentrations of soluble CD25 (soluble IL-2 receptor levels): 6,954 pg/mL (normal value of local laboratory reference: <2000). Given the direct correlation with increased T-cell and NK-cell activity, this last finding of elevated levels of soluble IL-2 as suggested by Mayama et al. [10], is a more specific diagnostic criteria for HLH [11]. We also performed perforin and CD107a testing, with both results not supporting impaired or absent NK-cell cytotoxicity. These tests are considered superior for screening patients for genetic HLH [12].

The diagnosis of primary HLH at the patient’s age would be very rare. Furthermore, the finding of normal NK-cell activity pleads against this diagnosis. Therefore only the presence of a *STXBP2* mutation was investigated, as NK-cell function testing in this mutation shows less severe impairment [13]. Given the remarkable lymphopenia at the patient’s admission (lymphocyte count of 420/mm^3^), the possibility of combined immunodeficiency could be taken into consideration if no increase in cell count after treatment was present. In fact, lymphocyte count remained at normal values outside the periods of disease activity.

When screening for infectious diseases, hepatitis B core antibodies were found to be positive. However, further testing confirmed an undetectable viral load.

Interestingly, high titer cardiolipin antibody IgG (50.8 U/mL) was found.

In our case, clinicians agreed that a viral infection was the most likely trigger of HLH in this patient, though one would expect remission after dexamethasone treatment and a more self-limiting course of the disease. One other possible scenario is the pregnancy itself inducing the disease. Some reports of pregnancy induced HLH describe a spontaneous recovery of the mother after a spontaneous abortion [14], see Table 1. Similar to the concept of pregnancy toxaemia in preeclampsia, altered foeto-maternal cell trafficking could lead to an uncontrolled auto-immune reaction causing HLH [14], [15], [16].

**Table 1:** Haemophagocytic lymphohistiocytosis during pregnancy: cases reported in the literature.

Author	Age (years)	Gestational age, weeks	Clinical presentation	Associated diagnosis	Ferritin, ng/mL	Bone marrow biopsy/aspiration	Treatment	Outcome	Mode of delivery
Mihara et al, 1999	32	16	Fever, pancytopenia	EBV	NA	+	corticosteroids, IVIg, acyclovir	Remission	35 weeks VD
Nakabayashi et al., 1999	30	21	Fever, arthralgia, pancytopenia,	PE, oligohydramnios, IUGR	7,240	+	AB, Ig, AT concentrate	Remission	29 weeks CS
Chmait et al., 2000	24	29	Lymphadenopathy, HELLP-like syndrome	EBV, necrotizing lymphadenitis	NA	+ (post-mortem)	AB, corticosteroids, acyclovir, Ig	Death	30 weeks CS
Yamaguchi et al. 2005	NA	NA	Genital herpes, fever, pancytopenia, hypertriglyceridemia	HSV2, temporary oligohydramnios	865.8	+	Corticosteroids, acyclovir, ciclosporin	Remission	37 weeks CS
Pérard et al. 2007	28	22	Fever, thrombocytopenia, anaemia	2-year history of SLE, PE, PPROM	1,500	+	AB, IVIg, corticosteroids	Relapse remission	30 weeks VD
Hanoka et al. 2007	33	23	Pancytopenia, hepatosplenomegaly, fever	BCNHL, oligohydramnios	587.6	+	Corticosteroids, CT, ASCT	Complete remission	28 weeks CS
Chien et al. 2009	28	23	Fever, skin rash, cytopenia, respiratory distress	-	136	+	AB, corticosteroids	Relapse remission	NA CS
Teng et al. 2009	28	23	Fever, anaemia, hepatosplenomegaly, thrombocytopenia	AIHA	8,926	+	Corticosteroids	Failure remission	29 weeks CS
Arewa et al. 2011	31	21	Fever, jaundice, abdominal pain, anaemia	HIV, malaria	NA	+	Antimalaria therapy, HAART	Remission	A term CS
Dunn et al. 2013	41	19	Rash, headache, fever, cytopenia	Still’s disease, IUGR	3,745	+	AB, corticosteroids	Remission	30 weeks CS (twin)
Hannebicque-Montaigne et al. 2012	29	21	Fever	SLE	>1,000	-	AB, corticosteroids, Ig	Remission	38 weeks VD
Shukla et al. 2013	23	10	Fever, hepatosplenomegaly, Pancytopenia	–	>2,200	+	Corticosteroids	Remission	Miscarriage
Mayama et al. 2014	28	20	Fever, pancytopenia	Parvovirus B19, foetal hydrops	1,269.2	+	Corticosteroids	Remission	37 weeks VD
Tumian and Wong 2015	35	38	Fever, HELLP-like syndrome	CMV	NA	+	Corticosteroids, cyclosporine, ganciclovir, AB	Death	38 weeks SC
Samra et al. 2015	36	16	Fever, pancytopenia, hepatosplenomegaly	–	4,000	−	AB, corticosteroids	Remission	NA VD
Giard et al. 2016	35	13	Lymphadenopathy, fever, pancytopenia	Kikuchi-Fujimoto lymphadenitis	4,567	+	AB, corticosteroids, etoposide	Death	NA miscarriage
Ota et al. 2016	26	23	SIRS, elevated ferritin	Pyogenic liver abscess	NA	+	–	Death	FDIU
He et al. 2017	27	30	Fever, pancytopenia, splenomegaly	EBV, NK/T-cell lymphoma	438.6	+	Corticosteroids, etoposide, rituximab	Death	30 weeks SC
Kerley et al. 2017	33	22	Dyspnoea, HELLP-like syndrome, hepatosplenomegaly	–	>500	+	Delivery, AB, corticosteroids, ciclosporin, etoposide, allogenic BMT	Failure, remission	22 weeks VD (foetal death)
Fernandez et al. 2017	20	24	Fever, epistaxis, respiratory failure	Tuberculosis PPROM, oligohydramnios	NA	+	AB, Ig, corticosteroids, ciclosporin, etoposide	Remission	29 weeks SC
Yildiz et al. 2017	36	29	Jaundice, cytopenia, fever	HIV	1,054.3	+	AB, corticosteroids	Remission	32 weeks SC
Parrott et al. 2019	28	18	Fever, splenomegaly, pancytopenia	SLE, IUGR	3,534	+	Corticosteroids, etoposide	Death	21 weeks FDIU
Parrott et al. 2019 (case 2)	37	24	Fever, jaundice, Rash	Acute liver failure, IUGR, CMV	8,110	−	Corticosteroids, etoposide	Remission	37 weeks VD
Sánches-Ato et al. 2019	23	27	Fever, rash, hepatomegaly	Steatohepatitis	1,345	+	AB, corticosteroids, etoposide, cyclosporine	Death	29 weeks FDIU
Our case	26	13	Fever, pancytopenia, hepatosplenomegaly	IUGR, anhydramnios	2,898	+	AB, corticosteroids, cyclosporine	Relapse, remission	27 weeks TOP

NA, data not available; EBV, Epstein Barr Virus; IVIG, intravenous immunoglobulin; VD, vaginal delivery; PE, pre-eclampsia; AB, antibiotics; IUGR, intrauterine growth restriction; Ig, immunoglobulins; AT concentrate, Antithrombin (III) concentrate; CS, Caesarean section; HSV2, Herpes simplex virus type 2; SLE, systemic lupus erythematosus; PPROM, premature rupture of membranes; BCNHL, B-cell non-Hodgkin lymphoma; CT, chemotherapy; ASCT, autologous stem cell transplantation; AIHA, auto-immune haemolytic anaemia; HIV, human immunodeficiency virus; HAART, highly active antiretroviral treatment; CMV, cytomegalovirus; SIRS, systemic inflammatory response syndrome; FDIU, foetal death *in utero*; BMT, bone marrow transplantation; TOP, termination of pregnancy.

Treatment of HLH may be hindered by diverse clinical course, high risk of treatment-related morbidity and disease relapse [17]. Offering therapy during pregnancy demands extra caution as some of the medications are associated with foetal toxicity. The current standard therapy consists of a decrescendo course of etoposide and dexamethasone, with or without intrathecal therapy [17].

Unfortunately there are no clinical practice guidelines on the management of pregnancy-related HLH [18], see Table 1. The main goal is to treat the underlying trigger and to lessen the gravity of the overactive immune system [19]. Use of etoposide in pregnancy has been reported to cause important side effects such as maternal and foetal bone marrow suppression [20, 21]. As a result, treatment with chemotherapeutic agents should be avoided during pregnancy. In this case, high-dose corticosteroids followed by addition of disease-modifying agents (e.g. ciclosporin) during disease reactivation was chosen.

## Take-home messages

In this reported case, HLH was fortunately recognized on time and treatment was rapidly initiated. Although the course of disease was very unfavourable to foetal viability, maternal survival was ensured. It is very important to keep the diagnosis of HLH in mind when persistent fever, cytopenia and a negative infection screen are present. Our case highlights the possible utility of H-score also in pregnancy. Marked hyperferritinaemia along with hepatosplenomegaly are crucial signs to raise suspicion. In the setting of pregnancy, obstetrical emergencies such as HELPP syndrome should always be ruled out. A multidisciplinary approach is essential to successful management of these complex cases.
